# Cytotoxic T lymphocyte lysis of HTLV-1 infected cells is limited by weak HBZ protein expression, but non-specifically enhanced on induction of Tax expression

**DOI:** 10.1186/s12977-014-0116-6

**Published:** 2014-12-14

**Authors:** Aileen G Rowan, Koichiro Suemori, Hiroshi Fujiwara, Masaki Yasukawa, Yuetsu Tanaka, Graham P Taylor, Charles RM Bangham

**Affiliations:** Section of Virology, Department of Medicine, Imperial College London, London, W2 1PG UK; Department of Bioregulatory Medicine, Graduate School of Medicine, Ehime University, and Ehime University Proteomedicine Research Center, Toh-on city, Ehime Japan; Graduate School and Faculty of Medicine, University of the Ryukyus, Okinawa, Japan

**Keywords:** HTLV-1, Retrovirus, Cytotoxic lymphocyte response, CTL, HBZ, Tax, HLA, ICAM-1, Fas

## Abstract

**Background:**

Immunogenetic evidence indicates that cytotoxic T lymphocytes (CTLs) specific for the weak CTL antigen HBZ limit HTLV-1 proviral load in vivo, whereas there is no clear relationship between the proviral load and the frequency of CTLs specific for the immunodominant antigen Tax. In vivo, circulating HTLV-1-infected cells express HBZ mRNA in contrast, Tax expression is typically low or undetectable. To elucidate the virus-suppressing potential of CTLs targeting HBZ, we compared the ability of HBZ- and Tax-specific CTLs to lyse naturally-infected cells, by co-incubating HBZ- and Tax-specific CTL clones with primary CD4^+^ T cells from HLA-matched HTLV-1-infected donors. We quantified lysis of infected cells, and tested whether specific virus-induced host cell surface molecules determine the susceptibility of infected cells to CTL-mediated lysis.

**Results:**

Primary infected cells upregulated HLA-A*02, ICAM-1, Fas and TRAIL-R1/2 in concert with Tax expression, forming efficient targets for both HTLV-1-specific CTLs and CTLs specific for an unrelated virus. We detected expression of HBZ mRNA (spliced isoform) in both Tax-expressing and non-expressing infected cells, and the HBZ_26–34_ epitope was processed and presented by cells transfected with an HBZ expression plasmid. However, when coincubated with primary cells, a high-avidity HBZ-specific CTL clone killed significantly fewer infected cells than were killed by a Tax-specific CTL clone. Finally, incubation with Tax- or HBZ-specific CTLs resulted in a significant decrease in the frequency of cells expressing high levels of HLA-A*02.

**Conclusions:**

HTLV-1 gene expression in primary CD4^+^ T cells non-specifically increases susceptibility to CTL lysis. Despite the presence of HBZ spliced-isoform mRNA, HBZ epitope presentation by primary cells is significantly less efficient than that of Tax.

**Electronic supplementary material:**

The online version of this article (doi:10.1186/s12977-014-0116-6) contains supplementary material, which is available to authorized users.

## Background

Human T lymphotropic virus type-1 (HTLV-1) persists in the host in dynamic equilibrium with the cytotoxic T cell response. Typically, virus-specific CD8^+^ cytotoxic lymphocytes (CTLs) in the peripheral blood of infected individuals are abundant and chronically activated. We have previously reported that circulating CTLs spontaneously kill HTLV-1-infected autologous CD4^+^ cells when co-cultured directly ex vivo [1], and the rate of CTL lysis of virus-expressing cells is inversely proportional to the proviral load [2,3], a clinical predictor of disease risk.

The program of viral gene expression in vivo plays an important role determining which CTL epitopes are protective in chronic infection. Two promoters in the HTLV-1 provirus direct transcription from the viral genome, one on each sense strand of the provirus. The plus stand encodes the viral transactivating protein Tax and other structural and non-structural proteins, and the minus strand encodes several splice variants of the HTLV-I basic leucine zipper factor (HBZ), which is biologically active as both RNA and protein [4,5]. Ex vivo, minimal plus-strand expression is detectable in infected peripheral blood mononuclear cells (PBMCs), whereas HBZ is persistently expressed [6]. Recent work in our laboratory has revealed that a typical infected individual possesses tens of thousands of clones of infected cells, each clone distinguished by its unique proviral integration site in the genome [7,8]. The genomic environment of the provirus influences both clone abundance in vivo and viral plus-strand reactivation ex vivo [9]; however, it is not known whether integration site influences expression of HBZ, or how HBZ expression interacts with Tax expression in naturally-infected cells.

The repertoire of viral epitopes exposed to CTL surveillance is determined by an individual’s human leukocyte antigen (HLA) genes, and HLA-A*0201 and Cw*08 are associated with reduced proviral load and disease risk in Kagoshima, Japan [10]. The ability of an individual’s HLA-alleles to bind peptides from HBZ has been shown to correlate inversely with proviral load and risk of HTLV-1-associated myelopathy/tropical spastic paraparesis (HAM/TSP) [11]. Despite its significant protective potential, the binding affinity of HBZ peptides to HLA class I molecules was found to be significantly weaker than that of peptides from Tax, and the frequency of HBZ-specific CD8^+^ T cells in peripheral blood was extremely low [11,12], although the IL-2 secreting HBZ-specific CD8^+^ T cells were more frequently detected in individuals with a viral load of below 1% of PBMCs [12]. In addition, HBZ protein is present at levels barely detectable by western blot; inefficient polyadenylation and transport of mRNA from the nucleus are thought to be responsible for this low expression [4,13–15]. Because of the low immunogenicity of HBZ, it has been difficult to directly test the ability of primary infected PBMCs to present HBZ to CTLs. Here, we therefore used HBZ- and Tax-specific CTL clones restricted by HLA-A*0201, which binds peptides from both HBZ and Tax with high affinity. The aims of the present study were to quantify the efficiency of presentation of Tax and HBZ epitopes to CTLs by primary, naturally-infected cells, and to test the hypothesis that the efficiency of CTL target formation is determined by virus-induced expression of specific host molecules on the cell surface.

## Results

**Figure 1:**
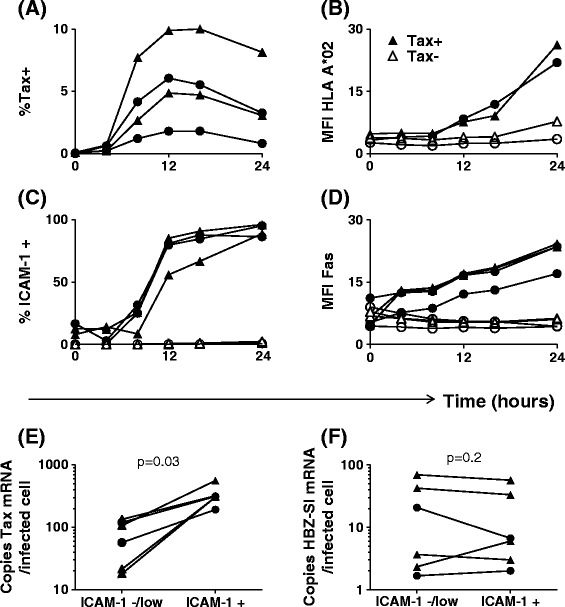
Expression of Tax, HBZ-SI, HLA-A*02, ICAM-1 and Fas by HTLV-1 infected primary CD4 ^**+**^ **cells. (A-D)** Upregulation of HLA-A*02, ICAM-1 and Fas occurs subsequent to the appearance of detectable levels of intracellular Tax protein. Purified CD4^+^ cells from four infected individuals (2 AC, circular symbols; 2 HAM, triangular symbols; two of which were HLA-A*02^+^) were cultured for 0-24 h. Surface molecules were stained with antibodies specific for HLA-A*02, ICAM-1, Fas, and CD4. Subsequently, cells were stained intracellularly with a Tax-specific antibody and analysed by flow cytometry. Values are expressed either as the percentage of CD4^+^ cells within the positive gate or as median fluorescence intensity on CD4^+^ T cells. **(E-F)** Expression of HBZ-SI and Tax mRNA by infected cells. CD8 depleted PBMC from six donors (2 AC, circular symbols; 4 HAM/TSP, triangular symbols) were incubated for 16 h to allow for Tax expression and ICAM-1 upregulation. Cells were subsequently sorted on the basis of ICAM-1 expression separating Tax^+^ (ICAM-1^+^) from Tax^**−**^ (ICAM-1^-/low^) cells. Exact copy numbers of HBZ-SI mRNA and Tax mRNA were quantified and expressed as number of copies per infected cell in each fraction. Statistics: ICAM^+^ vs. ICAM-1^low/−^, Wilcoxon matched pairs test, two tailed. See Additional file 6 for donor characteristics.

1. Plus strand gene expression is kinetically linked to upregulation HLA-A*02, ICAM-1, Fas and TRAIL-R1/2 on naturally infected CD4^*+*^*T cells*
  Initially, we characterised the phenotype of purified CD4^+^ T cells from HTLV-infected donors, both ex vivo and over 24 h culture in vitro. Using flow cytometry, we quantified expression of HLA-A*02, and a marker of viral plus-strand gene expression, Tax. In addition, we assayed expression of several surface molecules selected on the basis that they (1) are expressed by CD4^+^ T cells, (2) alter susceptibility to CTL lysis and (3) have been described to be either dysregulated in HTLV-1 infection, or induced by HTLV-1 proteins. At time zero, 100% of CD4^+^ T cells were HLA-A*02^+^. Tax protein was first detected after 8 h incubation (Figure 1A): subsequently, a population of HLA-A*02^high^, Fas^high^, ICAM-1^high^cells emerged (Figure 1B,C, D). In a cohort of ACs (n = 7) and individuals with HTLV-1-associated inflammatory disease (n = 8), multiparameter flow cytometric staining revealed that this population of cells co-expressed Tax, and Tax^+^CD4^+^ cells consistently expressed significantly higher levels of HLA-A*02, ICAM-1 and Fas than did Tax^−^CD4^+^ cells from the same donor (Figure 2). In comparison with ACs, donors with inflammatory disorders had a significantly higher proviral load (p = 0.009, Mann Whitney, two tailed) and correspondingly a higher frequency of Tax-expressing cells in the CD4^+^ population (p = 0.014, Mann Whitney, two tailed), however, there were no discernible differences in the phenotype of Tax-expressing cells between the two groups of donors (Additional file 1). Expression levels of HLA-A*02, ICAM-1 and Fas on Tax^+^ cells were strongly correlated with that of Tax protein in most individuals (Additional file 2). Similarly, we observed that TRAIL-R1/2 expression was moderately upregulated on Tax^+^CD4^+^ cells (Figure 2A), and, in HLA A*02^−^ donors, the median intensity of HLA-ABC expression on Tax^+^ cells was greater than Tax^−^ cells (Additional file 3).

**Figure 2:**
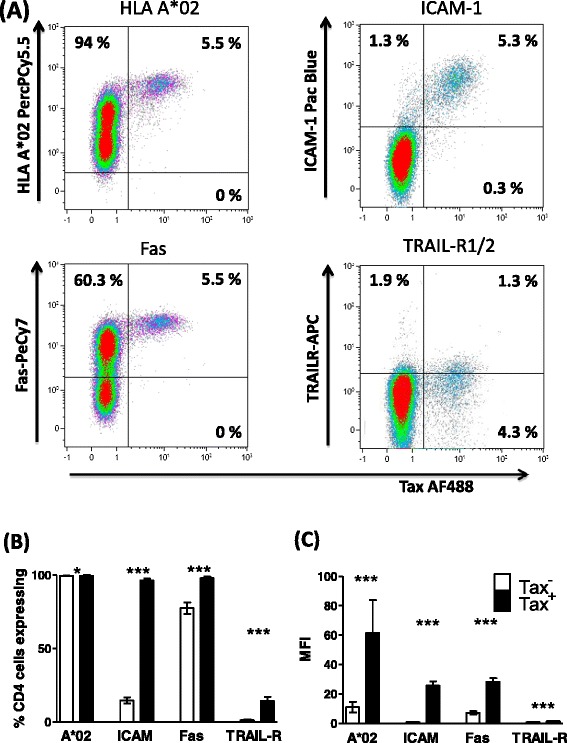
**Tax** ^**+**^ **CD4** ^**+**^ cells express elevated levels of HLA-A*02, ICAM-1, Fas, and TRAIL-R1/2. Purified CD4^+^ cells from 15 infected individuals (7 AC, 1 P, 7 HAM) were cultured for 24 h, after which the cell surface was stained with antibodies specific for HLA-A*02, ICAM-1, Fas, Trail-R1/2 and CD4. Subsequently, cells were stained for Tax protein and analysed by flow cytometry. **(A)** Staining of one representative individual’s CD4^+^ cells. Numbers indicate the percentage of cells in each quadrant. **(B)** Frequency and **(C)** intensity of surface protein expression on Tax^+^CD4^+^ (black bars) and Tax^−^CD4^+^ (white bars) for 15 individuals. Statistics: Tax^+^ vs. Tax^**−**^, Wilcoxon matched pairs test, two tailed; * denotes p < 0.02, *** denotes p ≤ 0.0002. Bars denote the mean ± SEM. See Additional file 6 for donor characteristics.
  These results show that plus-strand-expressing cells differ from uninfected cells in that they have a greater potential to present antigen (increased HLA class 1 expression), a greater ability to form a stable immunological synapse (increased ICAM-1 expression), and are primed to receive signals delivered via proapoptotic death receptors.
2. *HBZ-SI expression is maintained in cells which reactivate plus strand gene expression*
  Utilising the specific upregulation of ICAM-1 on cells which reactivated Tax expression, we enriched Tax^+^ cells and compared quantities of HBZ spliced isoform (HBZ-SI) and Tax mRNA with Tax^−^ cells from the same donor. Per infected cell, Tax expression in the ICAM^high^ fraction was significantly higher than observed in the ICAM^-/low^ fraction (Figure 1E); however, in contrast, levels of HBZ-SI mRNA did not differ between the two fractions (Figure 1F), indicating that over the course of this assay, induction of Tax expression did not significantly reduce levels of HBZ-SI mRNA in naturally infected cells.
3. *Tax*^*+*^*CD4*^*+*^*cells are resistant to Fas ligation-induced apoptosis*
  We tested whether Fas^high^ Tax^+^CD4^+^ cells were sensitive to apoptosis induction via the Fas pathway. While 5 h incubation with anti-APO-1/Protein A (100 ng-1 μg/ml) was sufficient to induce phosphatidylserine exposure on the cell surface in 70-80% of Jurkat T cells (Figure 3), both Tax-expressing and non-expressing primary cells (from infected and uninfected donors) were resistant to apoptosis induced in this manner. Thus, elevated Fas expression in the first 24 h of viral gene expression is unlikely to play a major role in CTL-mediated lysis of infected cells. Tax^+^CD4^+^ cells had a higher viability than Tax^−^CD4^+^ cells after 24 h in vitro culture (Figure 3), confirming that HTLV-1 plus-strand-expressing cells have a survival advantage in vitro, regardless of Fas/TRAIL-R1/2 expression.

**Figure 3:**
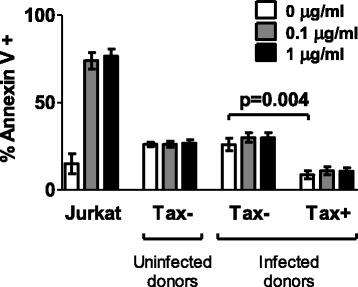
**Infected and uninfected primary CD4** ^**+**^ **T cells are resistant to Fas-induced apoptosis.** CD4^+^ T cells purified from nine HTLV-1-infected individuals (4 AC, 4 HAM, 1 uveitis) and six uninfected individuals were cultured overnight to allow Tax and Fas expression. Jurkat T cells were included in parallel as a positive control. Anti-Apo-1 and Protein A were added at the indicated concentrations, and cultures were continued for a further 5 hours. Apoptotic and necrotic cells were detected by a combination of Annexin V staining and Live/dead^TM^ viability dye. Statistical analysis was performed using the Wilcoxon matched pairs test, two-tailed. Bars denote mean ± SEM. See Additional file 6 for donor characteristics.
4. *Tax*^*+*^*CD4*^*+*^*cells are killed more efficiently than Tax*^*−*^*CD4*^*+*^cells when pulsed with peptide and coincubated with CTL specific for an irrelevant HLA-A*0201 restricted epitope
  To determine the net outcome of the altered phenotype of Tax^+^CD4^+^ cells, we directly quantified the ability of CTLs specific for an unrelated HLA-A*02-restricted Epstein Barr virus (EBV) epitope to kill CD4^+^ T cells from HLA-A*02^+^ HTLV-1-infected donors. CD4^+^ cells which had been cultured for 12 h to allow spontaneous viral gene expression were loaded with a peptide (GLCTLVAML) derived from the EBV BMLF-1 protein and mixed with a BMLF-1-specific CTL clone. After a further 12 h, we quantified the surviving Tax^+^ and Tax^−^CD4^+^ T cells. The results (Figure 4) show that the EBV-specific CTLs preferentially killed Tax^+^CD4^+^ cells.

**Figure 4:**
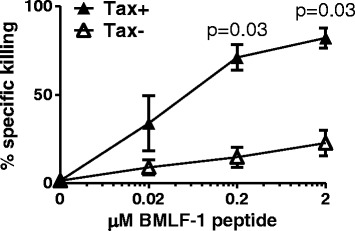
**Tax-expressing CD4** ^**+**^ cells are preferentially killed by CTL when loaded with HLA-A*0201 restricted peptide. Purified CD4^+^ cells from six HLA-A*02^+^ infected individuals (2 AC, 1 P, 3 HAM) were cultured for 12 h alone, to allow expression of viral proteins and any accompanying changes in cellular phenotype. BMLF-1 specific CTLs were introduced to the culture at an E:T ratio of 1:1 with and without the indicated concentration of BMLF-1 peptide. After a further 12 h incubation, the Tax expression by CD4^+^ cells was quantified by intracellular staining and flow cytometry. Percentage specific killing for each population and culture condition was calculated using the baseline frequency of Tax^+^CD4^+^ cells detected in the absence of specific peptide, and absolute CD4^+^ cell counts. Shaded symbols denote the frequency of Tax^+^ cells killed, open symbols denote the frequency of Tax^**−**^ cells killed for each donor. Statistical analysis was performed using the Wilcoxon matched pairs test, Tax^+^ vs Tax^**−**^, two-tailed. Bars denote mean ± SEM. See Additional file 6 for donor characteristics.
5. *Tax- and HBZ-specific CTL clones used in this study sensitively detect naturally processed epitopes*
  Before testing the relative levels of presentation of Tax and HBZ by naturally infected cells, we characterised the CTL clones used in this study. The Tax-specific CTL clone, derived from an infected donor, was itself infected with HTLV-1, but did not express detectable levels of Tax protein by flow cytometry (Additional file 4). CTL sensitivity was quantified by incubating a range of concentrations of peptide with HLA-A*02^+^ T2 cells in the presence of cognate CTLs (Figure 5A). At the saturating peptide concentrations both clones killed approximately 70% of peptide-loaded targets. The HBZ-specific CTL clone (HBZ-1) efficiently killed targets loaded with low peptide concentrations (EC50: 2 nM), and indeed was more sensitive than the Tax-specific CTL clone (Tax-1, EC50: 40 nM). Therefore, any observed reduction in the rate of killing of naturally-infected cells by the HBZ-specific clone was due to the level of epitope processing or presentation by target cells, rather than lower CTL efficiency. To confirm that the HBZ_26–34_ epitope is processed and presented naturally, we transfected cells with an expression plasmid encoding full-length HBZ (HBZ-IRES-GFP) or GFP alone [13]: HBZ-transfected cells were selectively killed by HBZ-1 CTLs (Figure 5B).

**Figure 5:**
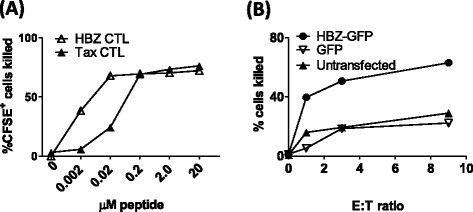
**HBZ-1 efficiently recognises both naturally processed and exogenously loaded HBZ**_**26–34.**_ **(A)** HBZ-1 detects its cognate epitope with greater sensitivity than Tax-1. CFSE-labelled T2 cells were incubated with a range of concentrations of the HBZ_26–34_ or Tax_11–19_ peptides for 1 h. Where indicated, CTL clones HBZ-1 or Tax-1 were added at an E:T ratio of 3:1. After 6 h incubation, cells were fixed, and the absolute number of CFSE^+^ cells present in each culture condition was determined by flow cytometry. These data are representative of three experiments performed in duplicate. **(B)** HBZ_26–34_ is processed and presented to CTLs. BLCL were transiently transfected with either an expression plasmid which encoded HBZ and GFP, or GFP alone. Cells were incubated for 18 h to allow gene expression, peptide processing and presentation, after which HBZ-1 CTLs were added at the indicated ratio. Absolute numbers of GFP-positive cells were quantified by flow cytometry. Results are representative of two experiments performed in triplicate.
6. *Primary Tax*^*+*^*CD4*^*+*^*T cells are killed by Tax-specific CTLs but not by HBZ-specific CTLs*
  CD4^+^ T cells from HLA-A*0201^+^ HTLV-1-infected individuals were cultured alone, or in the presence of HBZ- or Tax-specific CTL clones. Because of technical limitations in detecting HBZ protein in PBMC by flow cytometry, we used Tax protein staining as a surrogate to directly identify virus expressing cells. Tax expression was quantified after overnight incubation (Figure 6A), and a reduction in the frequency of Tax^+^CD4^+^ T cells was interpreted as lysis of Tax-expressing cells by CTLs. As an equivalent amount of HBZ-SI mRNA was detectable in both Tax^+^ and Tax^−^ primary infected cells from the same donor, we reasoned that any killing of HBZ- expressing cells would also lead to a detectable reduction in the frequency of Tax^+^ cells. After 24 h in vitro culture, we estimate that a median of 14% of infected cells were expressing detectable levels of Tax protein (range 7-71%).

**Figure 6:**
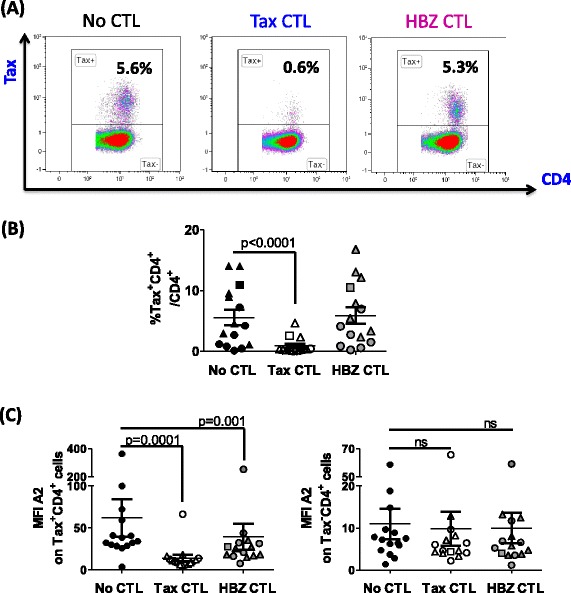
**Tax**_**11–19**_ **-specific but not HBZ**_**26–34**_ **-specific CTLs kill Tax-expressing primary cells efficiently.** Purified CD4^+^ cells from 7 AC (circular symbols), 1 P (square symbols), 7 HAM (triangular symbols), were cultured for 24 h alone, or in the presence of Tax-1 or HBZ-1 at an E:T ratio of 3:1. After incubation, surface and intracellular markers were stained and analysed by flow cytometry. **(A)** Tax staining for one representative individual; **(B)** frequency of Tax expression; **(C)** intensity of HLA-A*0201 expression for 15 individuals in the presence or absence of CTL selection. Statistics: Wilcoxon matched pairs test, two-tailed. NS = not significant. Differences observed were statistically significant regardless of whether the outlier (HEZ) was included or excluded. Bars denote mean ± SEM. See Additional file 6 for donor characteristics.
  After incubation with Tax-specific CTLs, on average 83% of Tax-expressing primary cells were lysed (Figure 6B). In addition, the median fluorescent intensity (MFI) of Tax staining was significantly reduced after incubation with Tax-specific CTLs (Additional file 2), consistent with our previous finding that cells expressing high levels of Tax protein were preferentially killed [2,3]. In contrast, HBZ-specific CTLs did not kill a significant number of Tax-expressing cells; nor was any reduction in Tax MFI observed. We conclude that presentation of HBZ_26–34_ by naturally-infected cells from HLA-A*A02^+^ individuals was not sufficient to efficiently trigger cytolytic functions at this E:T ratio.
7. Incubation with HBZ-specific CTLs selects against cells which express high levels of HLA-A*0201
  We quantified surface expression of HLA-A*02 after culturing primary CD4^+^ T cells alone, or in the presence of the HTLV-specific CTL clones. After co-incubation with either HBZ-specific or Tax-specific CTLs, there was a significant reduction in the median intensity of HLA-A*02 expression by Tax^+^CD4^+^ cells (Figure 6C). In contrast, the level of HLA-A*02 expression was unchanged on Tax^−^CD4^+^ cells when cultured in the presence of either of the CTL clones (Figure 6C).
8. *Competition between Tax*_*11–19*_*and HBZ*_*26–34*_*peptides*
  We hypothesized that Tax_11–19_ could inhibit presentation of HBZ_26–34_ by competing for binding to HLA-A*0201 (Figure 7A). At saturating concentrations of HBZ_26–34_ (2 uM), preincubation with Tax_11–19_ had no effect on lysis of BLCL by CTLs. However, when the concentration of HBZ_26–34_ was limiting (0.02- 0.2 uM), Tax_11–19_ could inhibit CTL detection of HBZ, but only when present in 10,000-fold molar excess. We also quantified the frequency of CD8^+^ T cells specific for the Tax_11–19_ and HBZ_26–34_ epitopes in each of the donors in this study. IFN-γ-producing Tax_11–19_ specific CD8^+^ cells were observed in all individuals, whereas no HBZ_26–34_ specific CD8^+^ cells were detected (Figure 7B).

**Figure 7:**
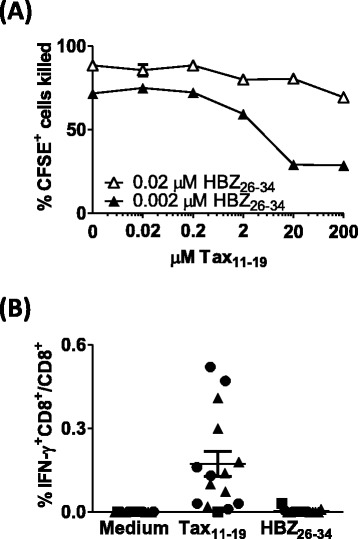
**Competition between Tax**_**11–19**_ **and HBZ**_**26–34.**_ **(A)** Tax_11–19_ inhibits presentation of HBZ_26–34_ under certain conditions. CFSE-labelled BLCL were incubated with a range of concentrations of Tax_11–19_ peptide for 1 h. HBZ_26–34_ peptide was subsequently added. After a further 1 h culture, HBZ-1 CTLs were added at an E:T ratio of 3:1. After 6 h incubation, cells were fixed, and the absolute number of CFSE^+^ cells present in each culture condition was determined by flow cytometry. These data are representative of two experiments performed in duplicate. **(B)** Recall CTL responses to HBZ_26–34_ and Tax_11–19._ PBMC depleted of CD4^+^ T cells were cultured either alone, or in the presence of 2 uM Tax_11–19_ or HBZ_26–34_. The frequency of IFN-γ producing cells was enumerated by Elispot. AC are represented by circular symbols; P, square symbols and HAM/TSP, triangular symbols.

## Discussion

We observed that viral reactivation altered expression of several molecules which modulate susceptibility to CTL mediated lysis: HLA-A*02, ICAM-1, Fas, and TRAIL-R1/2 were upregulated on the surface of unstimulated primary CD4^+^ T cells which expressed Tax. Whilst the absolute number of infected cells varied with proviral load, no significant differences in phenotype or susceptibility to lysis were observed between Tax-expressing CD4^+^ cells from ACs and those from individuals with HTLV-1-associated inflammatory conditions.

Whilst HTLV-1 p12 has been shown to direct degradation of MHC class 1 [16], this effect appears to be overridden by viral reactivation and Tax expression. The means by which HLA-A*02 expression is increased on Tax^+^CD4^+^ cells is unclear: possible mechanisms include MHC-peptide complex stabilisation by large quantities of high-affinity viral epitopes [17], or direct induction of MHC gene expression driven by virus proteins [18], T cell activation, or cytokines such as interferons and TNF-α [19]. ICAM-1 expression has been extensively studied in HTLV-1 infection [20]; both Tax and p12 can transactivate the ICAM-1 gene [21–23], and cross-linking of ICAM-1 enhances proviral expression [24]. In the virological synapse, the interaction of LFA-1 on uninfected cells with ICAM-1 on Tax^+^ infected cells induces cytoskeletal rearrangement and directional assembly and budding of viral particles [25,26]; the ICAM-1-LFA-1 interaction may also explain why HTLV-1-specific CTLs are preferentially infected with HTLV-1 [27]. In the immunological synapse, LFA-1 ligation enhances signalling from the T cell receptor in CD8+ T cells [28,29] lowering the activation threshold for effector functions. Since the absolute number of peptide-HLA complexes and the stability of the immunological synapse are integral factors in triggering CTL killing [30], these results suggest that Tax expression lowers the threshold for CTL-mediated lysis.

Fas ligand and TRAIL are contained within lytic granules of CTLs, and are exposed at the surface of CTL during degranulation, inducing apoptosis through initiation of the caspase cascade in target cells expressing the death receptors Fas and TRAIL-R. Ex-vivo primary T cells from uninfected donors are resistant to Fas-ligation-induced apoptosis [31]. We hypothesised that the elevated level of Fas expression by Tax^+^ cells might render them susceptible to Fas-mediated apoptosis: however, we observed that both Tax^+^ and Tax^−^ primary cells from infected donors were resistant to apoptosis induction with an anti-Fas antibody. Interestingly, Tax^+^ CD4^+^ cells actually survived in vitro more frequently than Tax^−^ cells from the same donor (Figure 3). There are conflicting reports in the literature whether Tax exerts pro- or antiapototic effects (reviewed in [32]), and although this subset of cells was identified by virtue of their expression of Tax protein, other viral proteins may be responsible for the observed enhanced viability in vitro. Indeed, a recent report indicates that HBZ can inhibit apoptosis induction by interfering with FoxO3A localisation and function [33]. It is conceivable that viral proteins have differing effects on cell survival, depending on the differentiation and activation status of the host cell. Our data on primary cells agree with observations that HTLV-1-infected T cell lines express high levels of Fas, but are resistant to Fas-L-induced apoptosis [34–36] and express FAP-1 (Fas inhibitor) [37].

Regardless of the observed resistance to Fas-induced apoptosis, Tax expression increased the sensitivity of peptide-loaded CD4^+^ cells to lysis by CTLs specific for an unrelated virus, EBV. Our observations were qualitatively identical in donors who were asymptomatic, and those with inflammatory conditions. Our findings are also in agreement with those of Kurihara et al. who showed that cultured PBMC from two patients with Adult T cell Leukaemia/Lymphoma (ATL) stimulated stronger responses than PBMCs from uninfected donors in a mixed lymphocyte reaction [38]. These changes in cellular phenotype were only observed after in vitro culture, and were not detected in fresh peripheral blood samples. Two possible explanations exist: either plus-strand gene expression occurs rarely in the peripheral blood, or virus-expressing cells are killed much more efficiently in vivo than in vitro.

Our choice of the HLA-A*0201 as a prototype MHC class I allele was influenced by several factors. Firstly, HLA-A*0201 strongly binds – and therefore efficiently presents – epitopes from both Tax and HBZ. Secondly, HLA-A*0201 is present in a high frequency of individuals in our cohort of donors, allowing us to test enough donors to generate statistically meaningful data. Thirdly, the availability of well-characterised high-avidity CTL clones facilitated direct comparison of the levels of presentation of each epitope. Finally, the availability of an HLA-A*02-specific antibody allowed us to quantify the level of expression of the exact molecule presenting the epitopes of interest (for further discussion, please see Additional file 5).

In contrast to Tax-specific CTLs, HBZ-specific CTLs were unable to kill large numbers of Tax-expressing cells, despite the fact that they expressed HBZ-SI mRNA and were highly susceptible to CTL lysis. HBZ-specific CTLs killed cells transfected with full-length HBZ, excluding a defect in processing or presentation of this epitope. Although HBZ-SI mRNA can be sequestered in the nucleus, this does not automatically exclude it from CTL surveillance, because recent evidence shows that peptides translated in the nucleus are targeted to the MHC class 1 presentation pathway [39]. A reduction in median fluorescence intensity of HLA-A*02 expression was observed after incubation with either the Tax-specific CTL clone or the HBZ-specific clone. This observation suggests that the HBZ epitope was effectively presented only by cells expressing the highest levels of HLA-A*02, and that the intensity of expression of HLA-A*0201 is more important than the level of Tax expression in determining whether an infected cell is susceptible to lysis by HBZ-specific CTL. We conclude that the level of presentation of HBZ by naturally-infected primary cells is limiting, even for an efficient (high-avidity) CTL clone. Further evidence of the poor immunogenicity of HBZ in vivo is the frequent lack of a detectable recall response to this epitope in HLA-A*02^+^ donors. These data are in agreement with the fact that, using HLA-A*02/HBZ_26–34_ tetramers, Suemori et al. could not detect CD8^+^ T cells specific for this particular epitope in ATL patients [40].

Affinity-dependent competition of peptides for binding to MHC class 1 is a potential confounding factor in this analysis. Tax_11–19_ has a very high affinity for the peptide-binding groove of HLA-A*0201, and so it could either displace or exclude other peptides with lower affinity. However, a 10,000-fold molar excess of Tax_11–19_ was required to inhibit detection of HBZ_26–34_ by the HBZ-specific clone. We conclude that such peptide competition is unlikely to materially impair presentation of HBZ epitopes in vivo.

The poor immunogenicity of HBZ for CTLs stands in contrast to the immunogenetic evidence [11] that CTL recognition of HBZ is associated with a reduction in proviral load and the risk of the inflammatory disease HAM/TSP. We propose that HBZ has evolved under CTL immune selection pressure to minimise its immunogenicity by reducing both the number and the class 1-binding affinity of peptide epitopes [11], and by restricting translation of HBZ mRNA, partly by retention in the nucleus [15]. HBZ-specific CTLs may exert their protective effect either by shaping the clonal population early in infection, or by applying a low but constant selection pressure on infected cells which express only the HBZ transcript in vivo.

## Conclusions

Tax protein is not only a strong immunogen, but also non-specifically increases susceptibility of the cell to CTL-mediated lysis. The net outcome of viral plus-strand gene expression will be determined by the balance between the resulting virus propagation (by both proliferation and de novo infection) and recognition by CTLs. The optimal strategy for HTLV-1 is to maintain persistent, low level expression of HBZ to promote proliferation, coupled with intermittent inducible expression of the highly immunogenic plus strand that is required for virion production. It is likely that CTLs specific for plus-strand antigens select and maintain a population of infected cells with integration sites compatible with this mode of gene expression.

## Methods

### Primary cells

All donors attended the National Centre for Human Retrovirology (Imperial College Healthcare NHS Trust, St Mary's Hospital, London), and donated blood having given informed consent in accordance with the Declaration of Helsinki, with approval of the UK National Research Ethics Service (09/H0606/106). PBMC were isolated from whole blood using Histopaque-1077 (Sigma) and cryopreserved in FCS (Invitrogen) with 10% DMSO (Sigma). Where indicated, CD4^+^ cells were isolated by positive selection using magnetic beads (Miltenyi Biotech). Unless otherwise stated, cells were cultured in RPMI-1640 (Sigma), 5% human AB serum (Invitrogen), supplemented with 2 mM L-glutamine, 50 U/ml penicillin, 50 μg/ml streptomycin and 20 μg/ml DNAse (Sigma). Genomic DNA was extracted using a DNeasy kit (Qiagen), and proviral load was estimated as described in Demontis et al. [41]. A 1/3 dilution series starting from 5 ng/ul genomic DNA was prepared, and the number of copies of Tax (primer pair: SK43 5’-CGGATACCCAGTCTACGTGT-3’ and SK44 5’-GAGCCGATAACGCGTCCATCG-3’) and beta-globin (primer pair: BG84F 5’-GCAAGGTGAACGTGGATG-3’ and BG84R 5’-TAAGGGTGGGAAAATTGACC-3’) present were quantified using FastSYBR mastermix (Life technologies) with the standard Fast SYBRgreen thermal cycle protocol on a QuantStudio 7 Flex real-time PCR system (Life technologies). A patient-derived infected CD4^+^ clone with a mapped single integrated provirus was used a reference standard [42]. In total, donors consisted of 16 AC (PVL 0.5-21%, mean 8.3%), 1 individual with polymyositis (P, PVL 21.6%), 1 individual with uveitis (UV, PVL 2.2%), 15 individuals with HAM/TSP (PVL 0.5-26%, mean 12.1%) and six uninfected individuals. To identify HLA-A*0201^+^ individuals, PCR-SSO HLA typing was performed by the Anthony Nolan Trust. Donor characteristics outlined in detail in Additional file 6.

### CTL clones

The Tax-specific CTL clone (Tax-1) was generated by limiting dilution cloning from an asymptomatic HTLV-1 carrier, and recognised the Tax_11–19_ epitope (LLFGYPVYV) in the context of HLA-A*0201. The HBZ-specific CTL clone (HBZ-1), which recognises the HLA-A*0201-restricted epitope HBZ-_26–34_ (GLLSLEEEL) [40], were generated in vitro from an uninfected, HLA-A*0201^+^ individual. HTLV-1 specific CTL clones were expanded by stimulation once per week with autologous gamma-irradiated B-LCL cells (5000 rad) with 1 uM cognate peptide (ThinkPeptides). An EBV-specific CTL clone (3H9) (a gift from Tao Dong), which recognises the HLA-A*0201-restricted epitope GLCTLVAML, was expanded by stimulation with gamma-irradiated (3000 rads) mixed allogeneic PBMC and 30 μg/ml phytohemagglutinin (Roche) once every two weeks. All CTL cultures were supplemented twice weekly with 100 IU/ml IL-2 (Promocell).

### HTLV-specific CTL lysis of primary infected CD4^+^ cells

CD4^+^ T cells from HLA-A*0201^+^ donors were cultured for 24 h either alone or in the presence of Tax-specific or HBZ-specific CTL clones at an effector:target (E:T) ratio of 3:1, and samples were then taken for flow cytometric analysis. Peptide-loaded HLA-A*0201-expressing T2 cells were used to monitor killing efficiency of CTLs on each day the experiment was performed. There was undetectable nonspecific killing of unloaded targets at the E:T ratio chosen (Additional file 2).

### Flow cytometric analysis

Cells were washed once in PBS, stained for 20 min with 0.25 μl/ml fixable live/dead blue viability stain (Molecular Probes), then washed with FACS buffer (PBS 7% normal goat serum). Surface molecules were stained for 20 min at RT with mAbs specific for cell-surface markers, as follows: CD4-Qdot605 (clone S3.5, Invitrogen), CD8-AF700 (LT8, Serotec), HLA-A*02-PerCPCy5.5 (BB7.2, BD Biosciences), TRAIL-R1/CD261-APC (DJR1, Biolegend), TRAIL-R2/CD262-APC (clone DJR-2-4, Biolegend), ICAM-1/CD54-Pacific Blue (HA58, Biolegend), Fas/CD95-PE (DX2, Biolegend). Cells were fixed and permeabilised using FoxP3 staining buffers (eBioscience), and stained with anti-Tax AF488 (LT-4) for 25 min at RT. Cells were washed, acquired using a BD LSR Fortessa, then analysed using Kaluza software (Beckman Coulter). Gating strategy is outlined in Additional file 7. We tested for correlation of markers by extracting data from the Tax^+^CD4^+^ population. Matched fluorescence intensity readings for each cell analysed were tested for correlation with spearman, using SPSS software.

### Quantification of viral RNA in Tax^+^ and Tax^−^ infected cells

PBMC depleted of CD8^+^ T cells were cultured for 16 h, harvested and stained with 0.4 ug/ml anti-ICAM-1-PE (Biolegend) then separated using anti-PE microbeads (Miltenyi Biotech) according to the manufacturers instructions. RNA was prepared using a “PARIS” RNA extraction kit and reverse transcribed using the “Vilo” kit (life technologies). HBZ-SI was amplified using the primer pair 5’-GGACGCAGTTCAGGAGGCAC-3’ and 5’-CCTCCAAGGATAATAGCCCG-3’, Tax with 5’-CCGGCGCTGCTCTCATCCCGGT-3’ and 5’-GGCCGAACATAGTCCCCCAGAG-3’, normalised to 18 s RNA detected using 5’-GTAACCCGTTGAACCCCATT-3’ and 5’-CCATCCAATCGGTAGTAGCG-3’ with FastSYBR mastermix on a QuantStudio flex7 system using the fast SYBRgreen thermal cycling protocol. Exact copy numbers was determined using a standard curve of the relevant target cloned into the pGEM-T-easy vector (Promega). In parallel, the proviral load of each fraction was estimated, and Tax protein and ICAM-1 expression was assayed by flow cytometry.

### Apoptosis assay

Jurkat T cells or primary CD4^+^ T cells were cultured at 1 × 10^6^ cells/ml for 16 h in RPMI 10% FCS, 20 μg/ml DNase. Anti-Apo-1 (anti-Fas) and Protein A (0.1-1 μg/ml, gift from Min-Li Weber) were added and the culture was continued for a further 5 h. Samples were washed in PBS and stained with live/dead blue stain. Cells were washed once in AnnexinV binding buffer (Biolegend) then stained with anti-CD4-PeCy5 (Beckman Coulter) and AnnexinV-PE (Biolegend) for 20 min at RT. The cells were washed and fixed for 20 minutes with 2% paraformaldehyde in AnnexinV binding buffer. Tax staining was performed as described above. Gating strategy is outlines in Additional file 8.

### CTL lysis assay using BLCL or T2 cells

HLA-A*0201^+^ autologous BLCL [40] or the TAP deficient lymphoblastic T2 cell line (a gift from Keith Gould) were used to estimate CTL sensitivity and as a positive control to monitor CTL performance in experiments assaying CTL lysis of primary CD4^+^ T cells. Cell lines were maintained in RPMI 1640 supplemented with 10% FCS, 2 mM L-glutamine, 50 U/ml penicillin, and 50 μg/ml streptomycin, seeded at a density of 1 × 10^5^ cells /ml and fed or split twice weekly as required. Cells were stained with CFSE (Molecular Probes), and 100,000 cells were loaded with peptide for 1 h before the addition of CTLs. Alternatively, 2 × 10^6^ BLCL were transfected with 2 μg HBZ-GFP expression plasmid or empty vector control (a gift from JM Mesnard [13]) using an Amaxa protocol (Kit C, program Z001, Lonza) 18 h prior to co-culture with CTLs. In every experiment, negative controls consisting of BLCL with no CTLs, and BLCL with CTLs but no peptide were included to quantify nonspecific killing. After 6 h incubation at 37°C, one volume of 4% paraformaldehyde was added to each tube, vortexed and incubated for 20 min to fix. Ten μl Countbright beads (Invitrogen) were added to each tube before acquisition. The number of cells surviving was calculated as follows:

$$ \#\mathrm{cells}\ \mathrm{in}\ \mathrm{t}\mathrm{ube} = \left(\#\mathrm{cells}\ \mathrm{collected}/\#\mathrm{beads}\ \mathrm{collected}\right) \times \mathrm{t}\mathrm{o}\mathrm{t}\mathrm{al}\ \#\mathrm{beads}\ \mathrm{added}\ \mathrm{t}\mathrm{o}\ \mathrm{t}\mathrm{he}\ \mathrm{t}\mathrm{ube}. $$

### CTL lysis assay using EBV-peptide loaded CD4^+^ T cells

CD4^+^ T cells were cultured alone at a density of 1 x 10^6^ cells/ml for 12 h. EBV-specific CTL clones were added at an E:T ratio of 1:1, with or without EBV BMLF-1 peptide. T cells were cultured for a further 12 h, then samples were taken for flow cytometric analysis. After 30 min incubation, cells were fixed by the addition of 1 volume 4% paraformaldehyde, and the cells were enumerated as described above. Tax, CD4 and CD8 were stained in parallel. Median nonspecific CTL killing in this assay was 24%.

### IFN-γ Elispot

Elispots were carried out as described in Kattan et al. [2] using an IFN-γ Elispot kit purchased from Mabtech.

## Additional files

Additional file 1:
**Comparison of AC and donors with HTLV-1 associated inflammatory disorders.**
Additional file 2:
**Extra data from CTL killing experiment, and correlations between expression of Tax, HLA A*02, ICAM-1 and Fas.**
Additional file 3:
**HLA- ABC staining and timecourse.**
Additional file 4:
**CTL clones do not express Tax protein.**
Additional file 5:
**Discussion of significance of HLA-peptide binding affinity.**
Additional file 6:
**Donor characteristics.**
Additional file 7:
**General gating strategy for flow cytometric analysis.**
Additional file 8:
**Gating strategy apoptosis assay.**

## Abbreviations

AC: Asymptomatic carrier
ATL: Adult T cell Leukaemia/Lymphoma
CTL: Cytotoxic T lymphocyte
EBV: Epstein barr virus
HAM/TSP: HTLV associated myelopathy / tropical spastic paraperesis
HBZ: HTLV-1 B-zip protein
HLA: Human leukocyte antigen
HTLV-1: Human T cell lymphotropic virus −1
MFI: Median fluorescence intensity
P: Polymyositis
PBMC: Peripheral blood mononuclear cells
SP1: Spliced isoform
