# Supplementary material for: Cytotoxic T lymphocyte lysis of HTLV-1 infected cells is limited by weak HBZ protein expression, but non-specifically enhanced on induction of Tax expression
Source: Retrovirology. 2014 Dec 14;11:116. doi: 10.1186/s12977-014-0116-6 (PMC4282740; doi:10.1186/s12977-014-0116-6)
Supplement: Additional file 1: — Comparison of AC and donors with HTLV-1 associated inflammatory disorders. [file 12977_2014_116_MOESM1_ESM.pdf]

Comparison of AC and donors with HTLV-1 associated inflammatory disorders

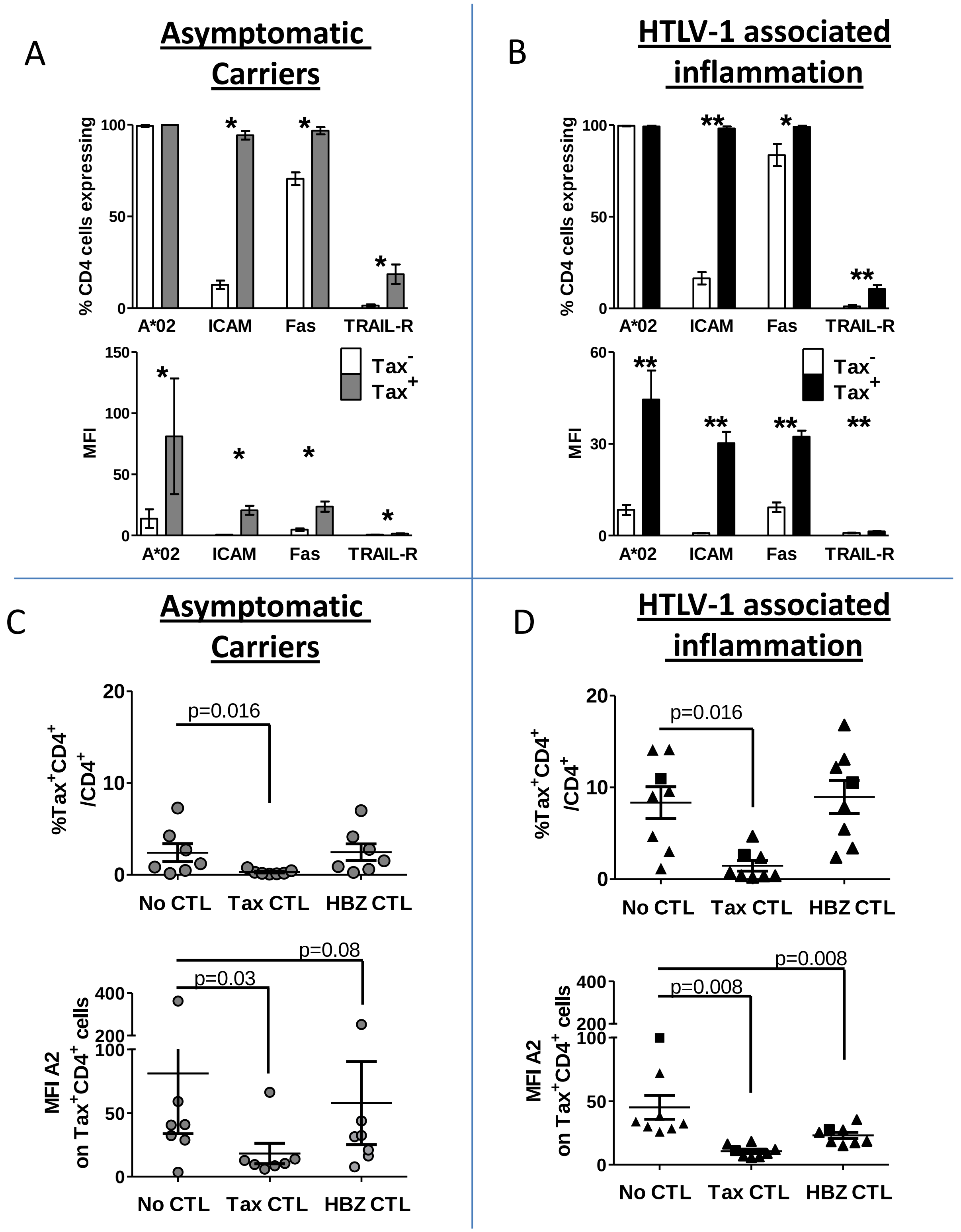

Legend: Phenotype of Tax + and Tax- cells from ACs (A), and individuals with HAM/TSP or polymyositis (B) All methods and further information can be found in the legend for figure 2. Lysis of CD4+ cells from ACs (C) and donors with HAM/TSP (triangles, D) or polymyositis (squares, D) by Tax-specific and HBZ-specific CTL. All methods and further information can be found in the legend for figure 6.
