# Supplementary material for: Cytotoxic T lymphocyte lysis of HTLV-1 infected cells is limited by weak HBZ protein expression, but non-specifically enhanced on induction of Tax expression
Source: Retrovirology. 2014 Dec 14;11:116. doi: 10.1186/s12977-014-0116-6 (PMC4282740; doi:10.1186/s12977-014-0116-6)
Supplement: Additional file 2: — Extra data from CTL killing experiment, and correlations between expression of Tax, HLA A*02, ICAM-1 and Fas. [file 12977_2014_116_MOESM2_ESM.pdf]

Extra data from CTL killing experiment, and correlations between expression of Tax, HLA A*02, ICAM-1 and Fas

(A)

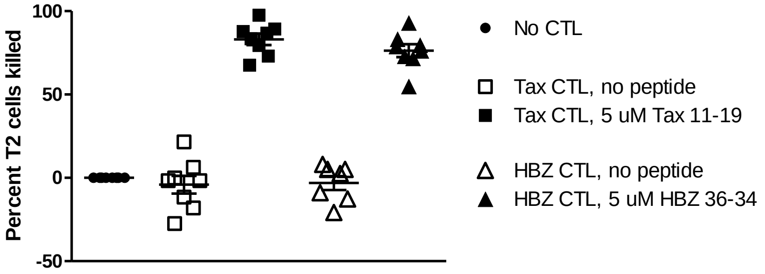

(B)

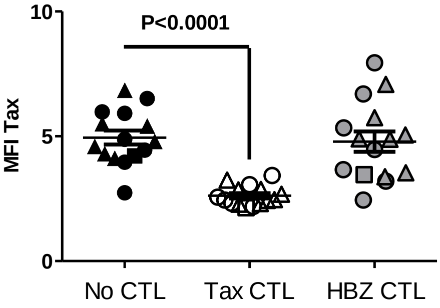

(C)

	Cultured alone		with HBZ-1 CTL		with Tax-1 CTL	
Donor	p	rs	p	rs	p	rs
HBT	**	0.605	NS		NS	
HCM	**	0.604	**	0.500	*	0.167
HDB	**	0.686	**	0.413	NS	
HDS	**	0.34	**	0.419	NS	
HEI	**	0.278	**	0.188	NS	
HEZ	**	0.207	NS		**	0.305
HFG	**	0.365	**	0.391	**	0.253
P4	**	0.477	**	0.250	**	0.147
TAA	**	0.258	**	0.105	NS	
TAC	**	0.174	**	0.150	NS	
TAQ	**	0.371	**	0.131	**	0.421
TAT	**	0.3	**	0.095	NS	
TBW	**	0.194	**	0.468	**	0.055
TCO	**	0.375	**	0.375	**	0.127
TCY	**	0.371	**	-0.084	NS	

(D)

	ICAM-1		Fas	
Donor	p	rs	p	rs
HBT	**	0.679	**	0.486
HCM	**	0.546	NS	
HDB	**	0.729	**	0.598
HDS	**	0.461	**	0.031
HEI	**	0.474	**	0.144
HEZ	**	0.465	NS	
HFG	**	0.514	**	0.177
P4	**	0.56	**	0.27
TAA	**	0.375	NS	
TAC	**	0.357	*	0.06
TAQ	**	0.234	NS	
TAT	**	0.411	**	0.125
TBW	**	0.57	NS	
TCO	**	0.481	**	0.255
TCY	**	0.468	**	0.099

Legend: (A) Tax and HBZ-specific CTL clones killed peptide loaded targets consistently and with undetectable nonspecific lysis of unloaded targets. (B) After CTL selection with Tax-specific CTL, the median intensity of Tax expression by surviving cells is significantly reduced. (C) Nonparametric bivariate correlation analysis between Tax and HLA-A*02 expression in the presence and absence of CTL selection. (D) Correlation analysis between Tax and ICAM -1 expression, and Tax and Fas expression in the absence of CTL selection. p= level of significance (*<0.05, ** <0.01) using spearman analysis. ns= no correlation. rs= correlation coefficient (spearman). All methods and further information can be found in the legend for figure 6.
