# Supplementary material for: Cytotoxic T lymphocyte lysis of HTLV-1 infected cells is limited by weak HBZ protein expression, but non-specifically enhanced on induction of Tax expression
Source: Retrovirology. 2014 Dec 14;11:116. doi: 10.1186/s12977-014-0116-6 (PMC4282740; doi:10.1186/s12977-014-0116-6)
Supplement: Additional file 3: — HLA- ABC staining and timecourse. [file 12977_2014_116_MOESM3_ESM.pdf]

HLA- ABC staining and timecourse

(A)

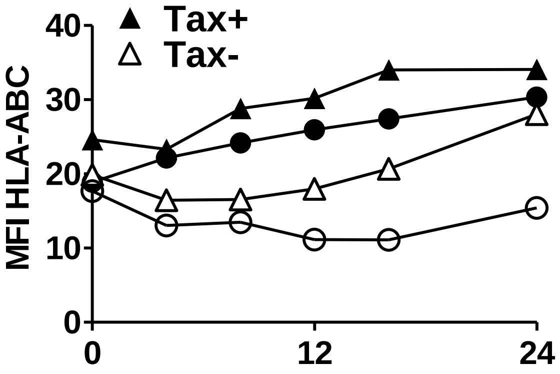

(B)

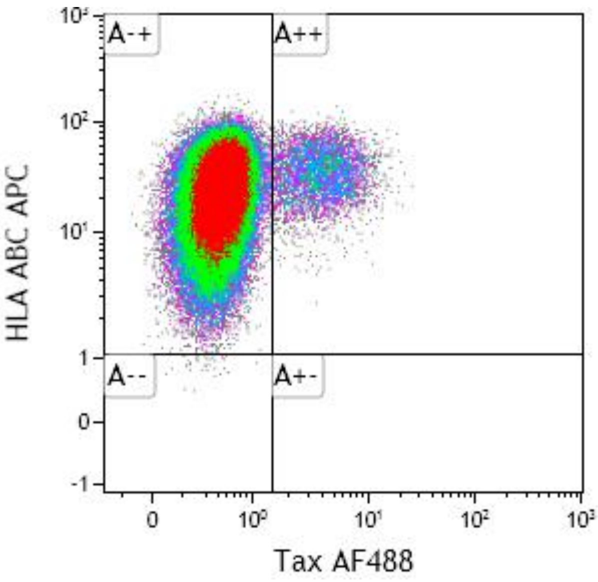

Legend: Timecourse (A) of HLA ABC expression by Tax^{+} and Tax^{-} cells over 24h culture in vitro. (B)Representative staining of Tax and HLA-ABC expression after 12 h culture in vitro. All methods and further information can be found in the legend for figure 1.
