# Supplementary material for: Cytotoxic T lymphocyte lysis of HTLV-1 infected cells is limited by weak HBZ protein expression, but non-specifically enhanced on induction of Tax expression
Source: Retrovirology. 2014 Dec 14;11:116. doi: 10.1186/s12977-014-0116-6 (PMC4282740; doi:10.1186/s12977-014-0116-6)
Supplement: Additional file 4: — CTL clones do not express Tax protein. [file 12977_2014_116_MOESM4_ESM.pdf]

CTL clones do not express Tax protein

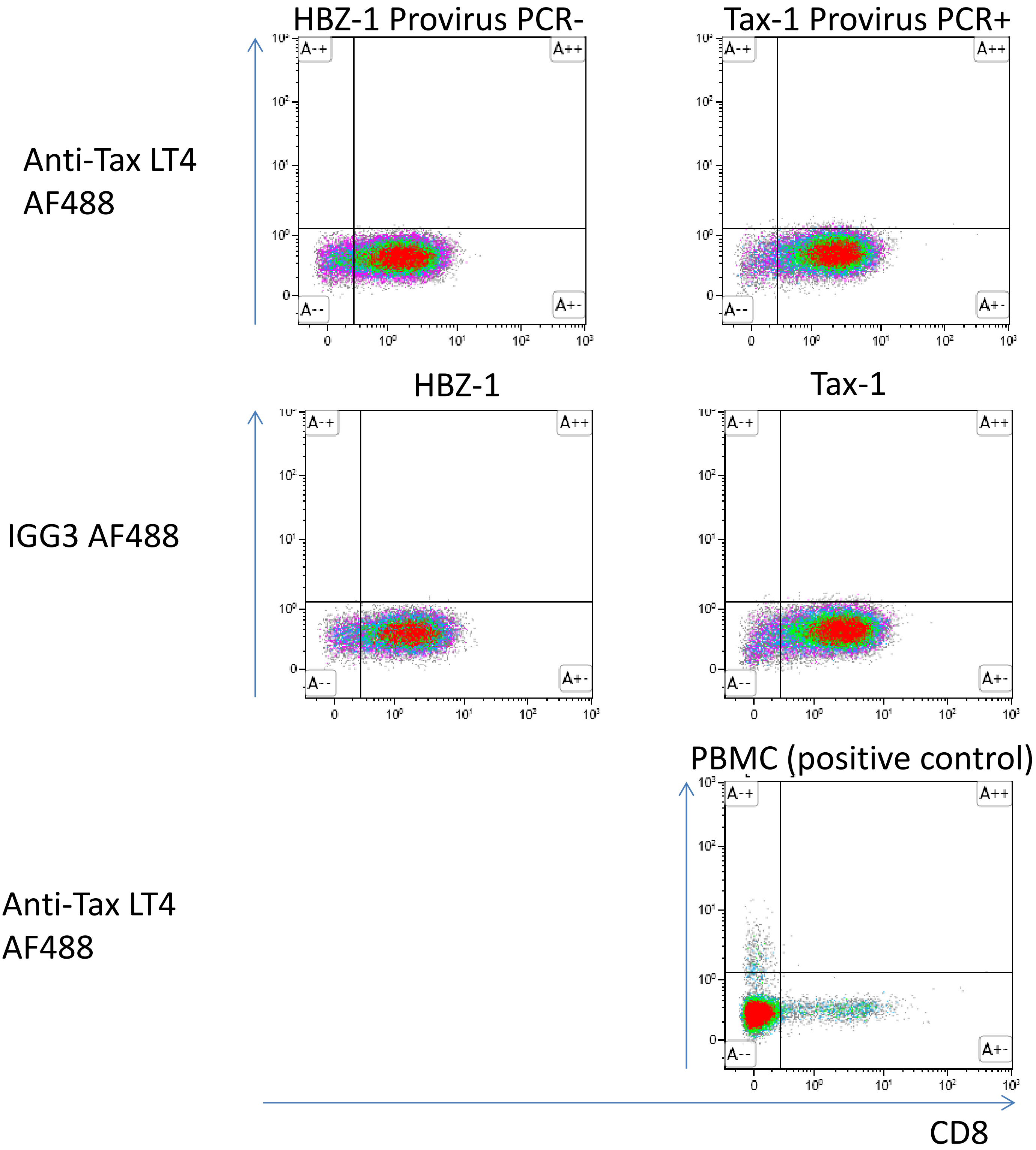

Legend: The Tax-1 CTL clone is infected with HTLV-1, but does not express Tax protein. CTL clones were stained with anti-CD8, anti-Tax, or an isotype control with an irrelevant specificity. PBMC from an HTLV-1 infected donor were cultured overnight and stained in parallel as a positive control.

All methods and further information can be found in the legend for figure 1.
