# Supplementary material for: Cytotoxic T lymphocyte lysis of HTLV-1 infected cells is limited by weak HBZ protein expression, but non-specifically enhanced on induction of Tax expression
Source: Retrovirology. 2014 Dec 14;11:116. doi: 10.1186/s12977-014-0116-6 (PMC4282740; doi:10.1186/s12977-014-0116-6)
Supplement: Additional file 5: — Discussion of significance of HLA-peptide binding affinity. [file 12977_2014_116_MOESM5_ESM.pdf]

Discussion of significance of HLA-peptide binding affinity

An equivalent ability to bind and present peptides from both Tax and HBZ is a critical consideration in the choice of HLA background for this study.

For example, consider HLA A*2402, which strongly binds a well characterised epitope in Tax. Peptides from HBZ are not predicted to bind to A*2402 with an affinity above the generally accepted threshold affinity for peptide binding (i.e. an IC50 of 500 nM). It is unlikely that any HBZ epitopes could be presented by this allele, thus would be an unsuitable allele to assess the CTL response to HBZ.

Of the 42 HLA alleles for which validated peptide binding algorithms exist, binding of the highest affinity peptide from Tax was equivalent or stronger than binding of the highest affinity peptide from HBZ. The only exception is A*3301, which was predicted to bind HBZ peptides strongly, and Tax peptides weakly [10]. Thus, HLAA*0201 is representative of individuals who possess both the potential to present HBZ peptides, and to generate a HBZ-specific CTL response.
