# Supplementary material for: Cytotoxic T lymphocyte lysis of HTLV-1 infected cells is limited by weak HBZ protein expression, but non-specifically enhanced on induction of Tax expression
Source: Retrovirology. 2014 Dec 14;11:116. doi: 10.1186/s12977-014-0116-6 (PMC4282740; doi:10.1186/s12977-014-0116-6)
Supplement: Additional file 6: — Donor characteristics. [file 12977_2014_116_MOESM6_ESM.pdf]

Donor characteristics

Donor	Experiment	Figure(s)	Genotype	PVL (%PBMC)	Age	Sex	Clinical status
HAI	timecourse	1	HLA-A*02-	17.12	74	F	Asymptomatic
HGP	timecourse	1	HLA-A*0201+	2.48	41	F	Asymptomatic
TCF	timecourse	1	HLA-A*02-	5.49	56	F	HAM/TSP
TCY	timecourse	1	HLA-A*0201+	8.97	55	F	HAM/TSP
HES	ICAM sort	1	HLA-A*02-	19.81	70	F	Asymptomatic
HHL	ICAM sort	1	HLA-A*0201+	20.99	25	F	Asymptomatic
TBP	ICAM sort	1	HLA-A*02-	19.52	73	M	HAM/TSP
TCH	ICAM sort	1	HLA-A*02-	10.36	61	F	HAM/TSP
TCK	ICAM sort	1	HLA-A*02-	6.50	64	F	HAM/TSP
TW	ICAM sort	1	HLA-A*02-	18.21	46	F	HAM/TSP
HBT	Lysis by HTLV CTL	2,6,7	HLA-A*0201+	1.07	72	F	Asymptomatic
HCM	Lysis by HTLV CTL	2,6,7	HLA-A*0201+	0.54	53	F	Asymptomatic
HDB	Lysis by HTLV CTL	2,6,7	HLA-A*0201+	1.20	54	F	Asymptomatic
HDS	Lysis by HTLV CTL	2,6,7	HLA-A*0201++	7.39	61	F	Asymptomatic
HEI	Lysis by HTLV CTL	2,6,7	HLA-A*0201+, *0205+	3.98	34	F	Asymptomatic
HEZ	Lysis by HTLV CTL	2,6,7	HLA-A*0201+, *0202+	14.69	55	F	Asymptomatic
HFG	Lysis by HTLV CTL	2,6,7	HLA-A*0201+	6.57	61	F	Asymptomatic
P4	Lysis by HTLV CTL	2,6,7	HLA-A*0201+	21.61	70	F	Polymyositis
TAA	Lysis by HTLV CTL	2,6,7	HLA-A*0201+	8.41	72	F	HAM/TSP
TAC	Lysis by HTLV CTL	2,6,7	HLA-A*0201++	20.24	66	F	HAM/TSP
TAQ	Lysis by HTLV CTL	2,6,7	HLA-A*0201+	6.91	84	M	HAM/TSP
TAT	Lysis by HTLV CTL	2,6,7	HLA-A*0201+	11.42	76	F	HAM/TSP
TBW	Lysis by HTLV CTL	2,6,7	HLA-A*0201+	26.71	68	M	HAM/TSP
TCO	Lysis by HTLV CTL	2,6,7	HLA-A*0201+	10.12	57	F	HAM/TSP
TCY	Lysis by HTLV CTL	2,6,7	HLA-A*0201+	8.97	54	F	HAM/TSP
U1	apoptosis induction	3	not tested	0	30	F	Uninfected
U2	apoptosis induction	3	not tested	0	33	F	Uninfected
U3	apoptosis induction	3	not tested	0	41	F	Uninfected
U4	apoptosis induction	3	not tested	0	42	M	Uninfected
U5	apoptosis induction	3	not tested	0	37	F	Uninfected
U6	apoptosis induction	3	not tested	0	76	M	Uninfected
HBE	apoptosis induction	3	HLA-A*0205+	12.69	77	F	Asymptomatic
HBX	apoptosis induction	3	HLA-A*02-	7.69	56	F	Asymptomatic
HDR	apoptosis induction	3	HLA-A*02-	1.73	35	F	Asymptomatic
HFE	apoptosis induction	3	HLA-A*0205+	13.27	68	F	Asymptomatic
TDO	apoptosis induction	3	not tested	0.49	46	F	HAM/TSP
TDT	apoptosis induction	3	HLA-A*02-	8.82	41	F	HAM/TSP
TDZ	apoptosis induction	3	not tested	19.37	55	F	HAM/TSP
TW	apoptosis induction	3	HLA-A*02-	18.21	44	F	HAM/TSP
UV1	apoptosis induction	3	HLA-A*02-	2.17	85	F	Uveitis
HBL	Lysis by EBV CTL	4	HLA-A*0201+	1.48	21	F	Asymptomatic
HEZ	Lysis by EBV CTL	4	HLA-A*0201+	14.69	57	F	Asymptomatic
P4	Lysis by EBV CTL	4	HLA-A*0201+	21.61	70	F	Polymyositis
TAQ	Lysis by EBV CTL	4	HLA-A*0201+	6.91	83	M	HAM/TSP
TCO	Lysis by EBV CTL	4	HLA-A*0201+	10.12	56	F	HAM/TSP
TCY	Lysis by EBV CTL	4	HLA-A*0201+	8.97	55	F	HAM/TSP

HLA-A*02^{-} denotes that no HLA-A*02 alleles were detected. Where HLA-A*02 alleles were detected, the high resolution genotype is specified. + = single copy of the allele, ++= two copies
