# Supplementary material for: Cytotoxic T lymphocyte lysis of HTLV-1 infected cells is limited by weak HBZ protein expression, but non-specifically enhanced on induction of Tax expression
Source: Retrovirology. 2014 Dec 14;11:116. doi: 10.1186/s12977-014-0116-6 (PMC4282740; doi:10.1186/s12977-014-0116-6)
Supplement: Additional file 8: — Gating strategy apoptosis assay. [file 12977_2014_116_MOESM8_ESM.pdf]

Gating strategy apoptosis assay

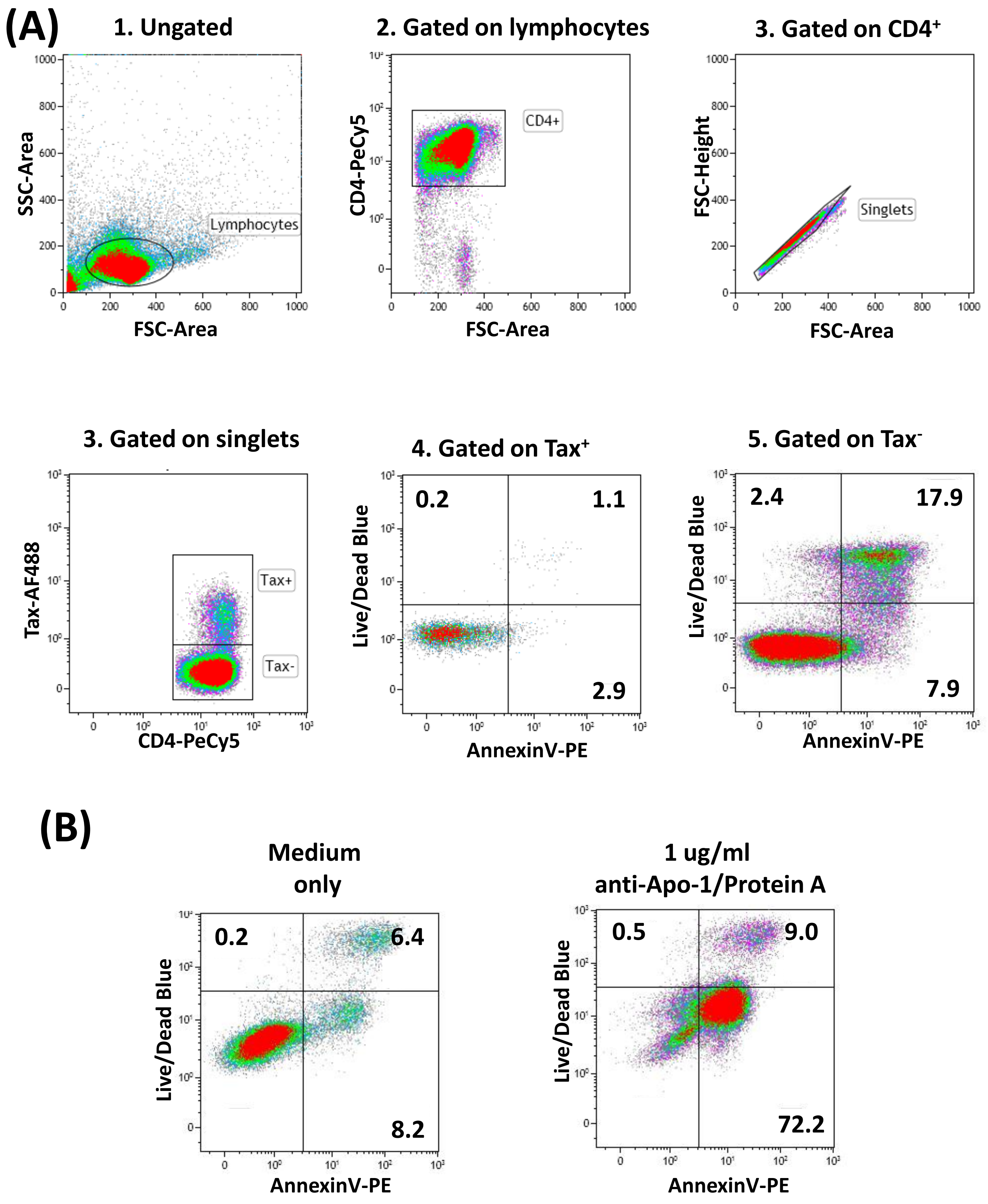

All methods and further information can be found in the legend for figure 3.
